# Interferon-α action in cytokine profile in eosinophilic nasal polyp cultures

**DOI:** 10.1016/j.bjorl.2019.08.010

**Published:** 2019-11-02

**Authors:** Júlio Cláudio Sousa, Renata Margarida Etchbehere, Eduardo Arthur Rodovalho Alves, Letícia Montes Stark, Eddie Fernando Cândido Murta, Márcia Antoniazi Michelin

**Affiliations:** a Universidade Federal do Triângulo Mineiro (UFTM), Faculdade de Medicina, Disciplina de Otorrinolaringologia, Uberaba, MG, Brazil; b Universidade Federal do Triângulo Mineiro (UFTM), Faculdade de Medicina, Disciplina de Patologia Especial, Uberaba, MG, Brazil; c Universidade Federal do Triângulo Mineiro (UFTM), Faculdade de Medicina, Instituto de Pesquisa em Oncologia (IPON), Uberaba, MG, Brazil

**Keywords:** Nasal polyposis, Cytokine, Interferon-α

## Abstract

**Introduction:**

Chronic rhinosinusitis is currently classified into two types: chronic rhinosinusitis without nasal polyps and chronic rhinosinusitis with nasal polyps. In the West, approximately 80% of chronic rhinosinusitis with nasal polyps cases are characterized by a predominantly eosinophilic cell infiltrate and a Th2 cytokine pattern.

**Objective:**

To evaluate the effect of Interferon-α on cytokine levels of the eosinophilic nasal polyp cell culture supernatant.

**Methods:**

Cell cultures were performed based on nasal polypoid tissue samples collected from 13 patients with eosinophilic chronic rhinosinusitis with nasal polyps. Polyps were considered eosinophilic according to the histopathological examination. Cell cultures were stimulated with 3000 IU of interferon-α. Before and after the stimulus, concentrations of Interferon-γ, tumor necrosis factor αand IL 2, 4, 6 and 10, using cytometric bead array, were assessed.

**Results:**

Cell samples from eosinophilic nasal polyps from 13 patients were included in the study. Twenty-four hours after interferon-α stimulation, eosinophilic nasal polyp culture supernatants showed significantly decreased IL-4 concentrations and increase in interferon-γ, IL-10 and IL-6 concentrations compared to controls. There were no significant differences in tumor necrosis factor -α and IL-2 concentrations.

**Conclusion:**

We demonstrated that interferon-α *in vitro* alters the pattern of cytokines in cell cultures of eosinophilic nasal polyps. Analysis of these alterations suggests that interferon-α promotes a rebalancing of inflammatory profiles in cell cultures, favoring the expression of Th1 and regulatory cytokines over Th2 cytokines.

## Introduction

Chronic Rhinosinusitis (CRS) is a complex and multifactorial disease, characterized by inflammation of the sinonasal mucosa for more than 12 weeks. Usually, two phenotypic presentations of CRS are differentiated, according to the presence of Nasal Polyps (NP): CRS without NP (CRSsNP) and CRS with NP (CRSwNP).^1^ Aspects related to cell infiltrate, cytokine pattern and tissue remodeling are distinct between CRSsNP and CRSwNP. CRSsNP is often associated with a Th1 response pattern, predominantly IFN-γ and TGF-β1, permeated by a predominantly mononuclear cell infiltrate. From the structural point of view, there is a predominance of fibrosis, goblet cell hyperplasia, basement membrane thickening and subepithelial edema.^2^ On the other hand, CRSwNP is commonly related to a Th2 response pattern, with a predominance of IL-4, IL-5 and IL-13, in addition to a predominantly granulocytic cell infiltrate, especially eosinophils. Regarding tissue remodeling, there is intense stromal edema with albumin deposition in CRSwNP, in addition to pseudocyst formation, basement membrane thickening, epithelial hyperplasia and glandular shortage.^3^ In fact, studies in western patients with CRSwNP show that approximately 80% of nasal polyps essentially have eosinophilic cell infiltrate.^4^, ^5^ However, studies involving the Asian population, especially from China, Korea and Japan, show that more than 50% of CRSwNP cases do not have eosinophilic predominance and, in some cases, neutrophils are the dominant cells.^6^, ^7^, ^8^ Therefore, it is evident that CRSwNP does not represent a homogeneous group regarding its immunological and histological characteristics. Since the CRSwNP phenotype does not presuppose a complete clarification of the underlying cell and molecular physio-disease, current studies have been moving towards the idea that CRSwNP comprises biological subtypes, or endotypes, which could be identified through biomarkers.^9^, ^10^

Eosinophilic CRSwNP usually has a more severe clinical picture, showing greater tomographic involvement (with special involvement of the ethmoid region),^11^, ^12^, ^13^, ^14^ higher association with comorbid asthma and worse clinical outcomes when submitted to surgical treatment.^15^, ^16^ Moreover, cases of eosinophilic CRSwNP commonly respond better to corticosteroid therapy, when compared to non-eosinophilic ones.^17^, ^18^ From the inflammatory point of view, eosinophilic CRSwNP is strongly influenced by Type 2 cytokines, such as IL-4, IL-5 and IL-13. These cytokines are produced by several cells, especially Th2-type cells, mast cells, and Type-2 innate lymphoid cells (ILC2s).^3^, ^10^

The therapeutic approach to eosinophilic CRSwNP is based on clinical and surgical treatments. Regarding clinical treatment, topical and oral corticosteroids have shown a high degree of efficacy and a enjoy strong recommendation in the literature. Surgical treatment of eosinophilic CRSwNP may be considered when there is no symptom improvement, despite maximum clinical treatment.^1^

Interferons (IFNs) comprise a set of glycoproteins that, due to their immunomodulatory, antiviral, and antiproliferative actions, have been widely used to treat a variety of chronic diseases, including multiple sclerosis, HCV infection, and certain types of cancer. They are also part of the therapeutic arsenal of eosinophilic diseases, such as Idiopathic Hypereosinophilic Syndrome (IHS), being an option in cases refractory to corticotherapy or, together with the latter, to reduce corticosteroid dosage.^19^, ^20^

Given the aforementioned facts, this study aims to evaluate the immunological action of Interferon-α, *in vitro*, against an essentially eosinophilic disease.

## Method

### Sample selection

A total of 13 patients with eosinophilic CRSwNP were selected from the years 2015 to 2017, who were followed at the Otorhinolaryngology Outpatient Clinic of Universidade Federal do Triângulo Mineiro (UFTM), of which 08 were males and 05 females, aged between 19 and 73 years.

During the selection process, patients older than 18 years with CRSwNP and tissue eosinophilia verified in the histological study of nasal polyps (more than 20 eosinophils per field of highest magnification) were included, according to the European Position Paper on Rhinosinusitis and Nasal Polyps 2012.^1^ Patients with CRSsNP and patients with CRSwNP with cystic fibrosis, allergic fungal rhinosinusitis or aspirin-exacerbated disease were excluded. Also excluded were patients with antrochoanal, sphenochoanal or ethmoid choanal polyps, as well as those with any unilateral polyps. None of the patients selected for the study had used topical or systemic corticosteroids within 30 days prior to the evaluation.

The study was submitted to the UFTM Research Ethics Committee, under approval number 2672.

### Overall study formatting

This is a prospective, experimental, self-paired study of 13 samples of eosinophilic nasal polyps. The study group consisted of cell cultures derived from eosinophilic nasal polyps, with application of interferon-α *in vitro*. For the control group, the same conditions were followed as those of the experimental group, but without interferon-α application.

All patients with CRSwNP who were willing to participate in the study, after clarifying doubts and signing the free and informed consent form, were scheduled to come to the UFTM Otorhinolaryngology Outpatient Clinic on a specific day. On such date, after undergoing a Paranasal Sinus Computed Tomography (PSCT) at the UFTM Imaging Department, the patients were referred to the Otorhinolaryngology Outpatient Clinic to have their clinical history assessed, and were submitted to otorhinolaryngologic examination, nasal endoscopy and nasal polyp biopsy. After the otorhinolaryngologic evaluation, the patients were referred to the UFTM Pulmonology Outpatient Clinic to undergo clinical evaluation and spirometry.

During the incisional biopsy of the nasal polyps, two fragments were collected from each patient. A fragment was placed in a conical plastic tube with formaldehyde and sent to the UFTM Anatomopathological Laboratory for histopathological analysis and cell infiltrate determination. The second fragment was placed in a sterile conical plastic tube with 0.9% saline solution and immediately sent to the UFTM IPON laboratory for cell culture.

### Paranasal sinus computed tomography (PSCT) and nasal endoscopy scores

The patients were submitted to PSCT without contrast, using a Toshiba Aquilion 64-channel CT scanner. The images, in 0.5-mm coronal and axial sections in volume and reconstruction of 5 mm in thickness, were analyzed by the researcher and classified according to the system proposed by Lund-Mackay.^21^ In this system, the paranasal sinuses (frontal, maxillary, anterior ethmoidal, posterior ethmoidal and sphenoidal) are scored as 0 (without opacification), 1 (partial opacification) and 2 (total opacification). The ostiomeatal complex also receives scores: grade 0 (unobstructed) and grade 2 (obstructed). Thus, considering the right and left sides of the analyzed images, the patients received a total score ranging from 0 to 24 points.

The endoscopy was performed in both nasal cavities using a Storz rigid fiber-optic endoscope, measuring 4 mm in diameter with zero angulation. The endoscopy quantification was based on the Lund-Kennedy proposal.^22^ According to this classification, the endoscopy is quantified as 0 (absence of polyps), 1 (polyps confined to the middle meatus) and 2 (polyps located beyond the middle meatus). Therefore, considering the right and left nasal passages, the patients' scores ranged from 0 to 4 points.

### Asthma characterization

All patients were evaluated at the UFTM Pulmonology discipline, which defined the presence or absence of asthma, according to the Guidelines of the Brazilian Society of Pulmonology and Physiology for Asthma Management - 2012.^23^

The following criteria were considered: clinical history (dyspnea, chronic cough, wheezing, chest tightness or discomfort, especially at night or early morning) and pulmonary function test (spirometry showing FEV1 / FVC < 75% with reversibility of at least 7% after salbutamol).

### Characterization of eosinophilia

The fragments used for the histopathological analysis were fixed in 4% diluted buffered formaldehyde, processed, embedded in paraffin and stained with the Hematoxylin-Eosin (HE) technique. The presence and amount of eosinophils in the biopsies were assessed simultaneously by two observers (a pathologist from the UFTM Pathology department and the researcher). The histological sections, measuring approximately 4-μm thick and stained with HE, were studied using a common optical microscope (OLYMPUS BX41®) and first under a 100-fold magnification for general evaluation. The eosinophils, present in subepithelial connective tissue, were counted according to a modification of the method proposed by Ruffoli et al.^24^ The eosinophils were quantified under a 400-fold magnification in 5 fields by averaging the number of eosinophils per field. Polyps with an average of > 20 eosinophils per high power field (HPF) were considered eosinophilic.

### Cell culture and interferon-alpha stimulation

Nasal polyp-derived cells were cultured at 37 °C in 2000 microliters of Roswell Park Memorial Institute (RPMI) 1640 medium containing 5% CO_2_, 10% fetal bovine serum, sodium bicarbonate, sodium pyruvate, 200 mμ of L-glutamine, 10,000 IU of penicillin, beta-2-mercaptoethanol and 10 mg/mL of streptomycin (complete medium marketed by Sigma).

The polyp fragment to be cultured was placed on a petri dish and agitated using anatomical tweezers and number 21 scalpel blades. The dispersion product was washed in the petri dish with incomplete RPMI medium and transferred to a 50 mL conical plastic tube. Incomplete RPMI medium was then added to the latter, up to a volume of 15 mL. The tube was then centrifuged at 4 °C at a speed of 2100 rpm for 10 min under refrigeration. Two centrifugation cycles were performed. Between the two, the precipitate was washed with incomplete RPMI medium. At the end of the last centrifugation, the excess solution was carefully removed, and the precipitate was resuspended in 8 mL of the solution. Afterwards, a Neubauer chamber was used for cell counting per milliliter (mL) of solution. Once known the amount of cells / mL of the solution, 10^6^ cells were seeded along with complete RPMI medium in a culture dish containing six wells. Each well totaled a volume of 2 mL. The cultures were then preserved in the incubator. All procedures were performed under sterile conditions using a laminar flow hood, except for cell counting.

Twenty-four hours after culture incubation, 3000 IU of interferon-α were applied to 4 wells, which were considered the experimental group. Interferon-α was not added to the remaining 2 wells, which comprised the control group. All samples were analyzed in duplicate.

After the aforementioned cytokine was applied, the cultures were returned to the incubator, considering this moment as time zero. Twelve hours after interferon-α was applied, the supernatant from the 2-well culture of the experimental group was collected, which was then called the 12 -h experiment. The cultures were returned to the incubator again.

Twenty four hours after the introduction of interferon-α, the supernatant was collected from the other 2 wells of the experimental group, which were classified as the 24 -h experiment. At this time, the supernatant from the two wells that did not undergo the action of interferon-α (control group) was also collected. All collected supernatant samples were stored in Eppendorf flasks and kept in the freezer at −80 °C.

Subsequently, it was analyzed in its entirety by Cytometric Bead Array (CBA), through which the concentration of interleukins IL-2, IL-4, IL-6, IL-10, TGF-β e IFN-γ was verified.

### Measurement of cytokines by cytometric bead array (CBA)

The supernatant samples collected from eosinophilic nasal polyp cell cultures were submitted to cytokine measurement using Cytometric Bead Array (CBA) (BD™ Biosciences, San Diego, CA). The CBA Th1/Th2 Cytokine Human Kit II measured six cytokines, representing a broad spectrum of mediators, among them interleukins 2, 4, 6 and 10, Tumor Necrosis Factor (TNF) and Interferon-γ (IFN-γ). The analyzed samples processing followed the manufacturer's instructions. The readings were made using a BD™ FACSCalibur® flow cytometer close to the standard curve, to determine reference concentrations as well as quality control. The software used for these analyses was FCAP Array Cytometric Bead Array version 1.4 (BD Biosciences, San Jose, CA), which works with an automated analysis system. According to the manufacturer's recommendations, all obtained standard curves followed the threshold for cytokine measurement with 99% accuracy, a condition that is absolutely required for the beginning of the analyses.

### Statistical analysis

There was no statistical calculation to define sample size, which was defined by accessibility. The results regarding cytokine measurement, once obtained, were placed into a database using Microsoft Access 2000® and evaluated using the GraphPad Prism 5 program for statistical study. The statistical comparison was performed by nonparametric analysis using Wilcoxon’s test. The tests were considered significant when the probability of rejection of the hypothesis was less than 5% (*p* < 0.05).

## Results

Thirteen patients participated in the study, as they met the inclusion criteria determined for the research. Patient age varied from 19 to 73 years, with a mean of 53 years. Among them, 8 patients were males (61%) and 5 females (39%), and 46% had asthma. The Lund-Mackay score of the tomographic assessment ranged from 12 to 22 points (mean = 17) for a total of 24 points. The endoscopy of the nasal cavities showed a Lund-Kennedy score ranging from 3 to 4 points (mean = 3.69) for a total of 4 points (Table 1).

**Table 1:** Sample characteristics according to age, gender, presence of asthma, Lund-Mackay and Lund-Kennedy.

Case	Age	Gender	Asthma	Lund-Mackay	Lund-Kennedy
1	72	F	Positive	22	4
2	42	M	Negative	12	3
3	69	M	Positive	20	4
4	68	F	Positive	22	4
5	53	M	Negative	14	4
6	35	F	Positive	14	3
7	54	M	Negative	15	4
8	73	F	Negative	16	4
9	50	M	Positive	18	4
10	19	F	Negative	14	4
11	44	M	Positive	22	3
12	58	M	Negative	16	4
13	52	M	Negative	17	3

**Figure 1:**
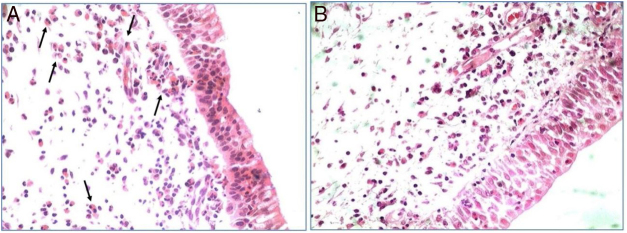
Hematoxylin-eosin stained nasal polypoid tissue. Eosinophilic infiltration, indicated by arrows, predominant in eosinophilic CRSwNP patients (A). Inflammatory infiltrate without eosinophilic predominant in patients with non-eosinophilic CRSwNP (B) (400×).

All polypoid tissue sample used in the study showed intense eosinophil infiltrate at the histopathological analysis (more than 20 eosinophils/HPF). This finding was used as a criterion to classify these nasal polyps as eosinophilic (Fig. 1).

**Figure 2:**
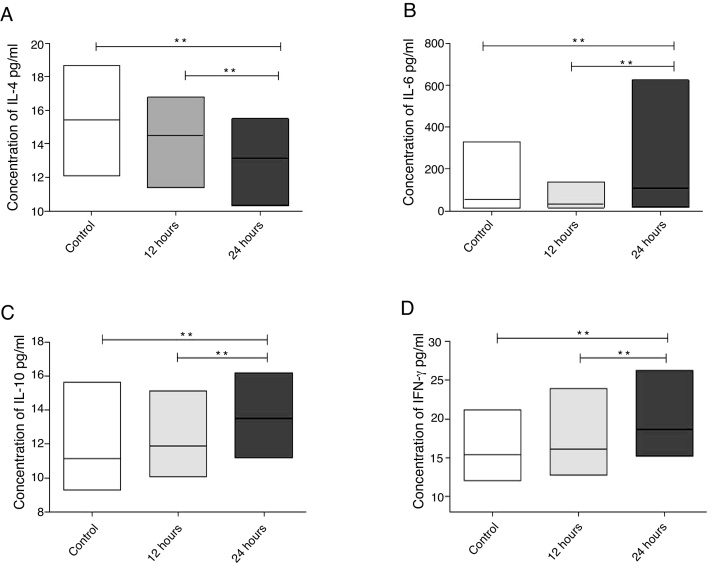
Cytokine levels in cell culture supernatants of eosinophilic nasal polyps after 12 and 24 h of IFN-a stimulation. Results were expressed as median, maximum and minimum concentration values (pg/mL) of IL-4 (A), IL-6 (B), IL-10 (C) and IFN-γ (D) cytokines. Results were analyzed using the nonparametric Wilcoxon test. The observed differences were considered significant when *p* < 0.05 (5%).

When evaluating the concentration of interleukins in the eosinophilic nasal polyp cell culture supernatant, a significant decrease in IL-4 concentration (*p* = 0.0078) was observed, when compared to controls 24 h after exposure of cultures to IFN-α action. It was also observed that a reduction in IL-4 concentration was significantly more intense in cultures evaluated 24 h after IFN-α stimulation, when compared to cultures evaluated 12 h after it (*p* = 0.0039) (Fig. 2A).

Regarding IL-6 (*p* = 0.0186), IL-10 (*p* = 0.0039) and IFN-γ (*p* = 0.0039), eosinophilic nasal polyp cell cultures showed a significant increase in their concentrations 24 h after IFN-α action, when compared to controls. It was also observed that the increase in the concentration of these cytokines was significantly more relevant in cultures evaluated 24 h after IFN-α stimulation, when compared to cultures evaluated 12 h after it (*p* = 0.0039 for IL-6, *p* = 0.0091 for IL-10 and *p* = 0.0269 for IFN-γ) (Fig. 2 B, C and D).

Regarding interleukins IL-2 and TNF-α, there was no significant difference in their concentrations when compared to controls after culture stimulation with IFN-α (data not shown in the charts). Table 2 shows the data regarding the median, maximum and minimum values of the assessed interleukins.

**Table 2:** Median, maximum and minimum values of interleukin levels measured in cell culture supernatants of eosinophilic nasal polyps.

	Control	12 h	24 h
IL-4	15.80 (12.10‒18.70)^a^	15.30 (11.40–16.80)^b^	13.20 (10.30–15.50)^a^, ^b^
IL-6	29.20 (12.20–331.50)^a^	20.90 (14.50–138.30)^b^	32.90 (17.90 – 625.90)^a^, ^b^
IL-10	10.70 (9.30–15.60)^a^	11.50 (10.10–15.10)^b^	13.80 (11.20–16.20)^a^, ^b^
IFN-γ	14.70 (12.10–21.20)^a^	15.60 (12.80–23.90)^b^	17.90 (15.20–26.30)^a^, ^b^
IL-2	13.80 (10.90–128.50)	13.10 (10.60–61.10)	12.95 (10.60–15.30)
TNF-α	12.00 (9.10–104.80)	12.20 (9.00–61.70)	11.30 (9.50–14.70)

a *p* < 0.05 for the comparisons between control and 24 h.

b *p* < 0.05 for the comparisons between 12 and 24 h.

## Discussion

Current knowledge about CRSwNP allows us to state that this disease represents an essentially inflammatory disease of unknown etiology. With the advancement of the understanding of the molecular and cell biology structure involving CRSwNP, it has become evident that the phenotypic diagnosis of CRSwNP encompasses a variety of distinct inflammatory pathways. The tendency, at the moment, is to individualize these pathways through the use of biomarkers, aiming to know the several CRSwNP endotypes.^25^

Studies have shown that in the West, most CRSwNP patients have an endotype characterized by a predominantly eosinophilic cellular infiltrate and an immunological profile, especially Th2 cytokines. These cytokines play an important role in the individualization of CRSwNP endotypes, as they participate in the adaptive immune response to exogenous factors and influence the local tissue environment by recruiting several cell types.^26^

The intensity of eosinophilic infiltration in the polypoid tissue of patients with CRSwNP characterizes the disease as eosinophilic or non-eosinophilic, and it has prognostic implications. Eosinophilic CRSwNP is generally associated with higher clinical and radiological severity and higher risk of asthma and polyposis recurrence after surgical treatment.^27^ Aslam et al. evaluated disease severity in 53 patients with CRSwNP. In patients with eosinophilic polyps (eosinophils >10 / HPF), the authors found an average score of 13.5/24 for the Lund-Mackay index (radiological involvement) and 2.8 / 4 for the Lund-Kennedy scoring system (nasal endoscopy involvement).^17^ In another study, Wang et al. also observed high rates of radiological (Lund-Mackay of 14.42 / 24) and nasal endoscopy involvement (Lund-Kennedy of 3.59 / 4) in patients with eosinophilic polyps (eosinophils >5 / HPF).^28^ According to the literature, the patients in this study also showed significant Lund-Mackay index (mean of 17/24) and Lund-Kennedy score (average of 3.69 / 4). Another finding of clinical importance was that approximately half of the patients in the sample (46%) had asthma. In fact, the association between asthma and CRSwNP is already known and, from a histopathological point of view, both share some alterations, such as eosinophilic inflammation, epithelial damage and thickening of the respiratory mucosa basement membrane.^29^ In a study by Hopkins et al., of 2176 patients with CRSwNP submitted to surgical treatment, 37.5% had asthma.^30^ Based on the analysis of these data, it can be inferred that the patients in the present study showed a clear agreement with the literature reports on other patients with eosinophilic CRSwNP, from the clinical, radiological and histopathological point of view.

The treatment of eosinophilic CRSwNP is still a challenge. Many patients show unsatisfactory disease control, despite maximum clinical and surgical treatment. Studies have shown that patients with eosinophilic CRSwNP have worse clinical outcomes and a greater tendency for polyposis recurrence after surgical treatment, when compared to non-eosinophilic ones.^31^ To date, corticosteroids, in either topical or systemic presentations, represent the options with the highest levels of evidence in the treatment of eosinophilic CRSwNP.^1^ However, as it is a disease with a chronic evolution, the frequent use of corticosteroids, especially in the systemic form, often results in undesirable side effects.^32^

Thus, given the limitations of the treatments available for eosinophilic CRSwNP and the current knowledge about the pathophysiology of the disease, therapeutic alternatives have recently been sought linked to the molecular basis of this disease. From this perspective, biological therapies using monoclonal antibodies directed against of biomarkers Th2 inflammatory response have gained relevance. The highlights include omalizumab (anti-IgE), reslizumab (anti-IL-5), mepolizumab (anti-IL-5) and dupilumab (anti-IL-4α receptor).^33^

Interferon-α (IFN-α) is a therapeutic option in eosinophilic syndromes, such as Idiopathic Hypereosinophilic Syndrome and Churg-Straus Syndrome. The clinical benefits of IFN-α in these diseases are supported by *in vitro* studies, which demonstrate the action of this cytokine on eosinophilia. Eosinophil incubation with IFN-α inhibits the release of eosinophil-derived neurotoxin and the eosinophil cationic protein, when they are stimulated with IgA and IgE.^34^ By promoting the development of Th1 lymphocytes, IFN-α can inhibit eosinophil differentiation and migration through the effects of IFN-γ.^35^ Finally, it has been shown that IFN-α inhibits IL-5, GM-CSF and IL-13 gene expression, important cytokines in eosinophil migration, proliferation, activation and survival.^36^

More recently, the use of IFN-α in the treatment of atopic diseases with a Th2 profile has been reported. In the case of asthma, an immunopathological disease that shares similarities with eosinophilic CRSwNP, it has been documented that clinical use of IFN-α improves lung function, reduces daily corticosteroid dose for symptom control, and decreases patients’ visits to emergency hospital units.^37^

In the field of otorhinolaryngology, IFN-α is rarely mentioned as a therapeutic option. There are no reports in the literature of the treatment of eosinophilic CRSwNP with IFN-α. Recently, Neff et al. reported the use of pegylated IFN-α in 8 patients with eosinophilic otitis media. The authors observed a satisfactory clinical response, with complete resolution of otorrhea, normalization of the middle ear mucosa and discontinuation of corticosteroid therapy in 50% of the cases.^38^ Considering these facts, the aim of the present study was to investigate *in vitro* whether IFN-α alters the cytokine profile of eosinophilic CRSwNP, which is known to have a predominant Th2 pattern.

In this study, it was found that the exposure of eosinophilic polyp cell cultures to IFN-α significantly decreased IL-4 concentration. This is an important Th2 cytokine produced by eosinophils, basophils, mast cells, NK cells, and Th2 cells. Some of its functions involve stimulating the differentiation of virgin T-lymphocytes into Th2 lymphocytes and being an important cofactor in the prevention of activated T cell apoptosis. Moreover, IL-4 is the main cytokine responsible for B lymphocyte immunoglobulin class switch to the IgE phenotype. IL-4 also participates in eosinophil recruitment by increasing VCAM-1 expression in endothelial cells.^39^ Therefore, knowing of the importance of IL-4 in the construction of a Th2 inflammatory environment, its reduction after stimulation with IFN-α suggests that it has the potential to negatively modulate Th2-type inflammation in eosinophilic CRSwNP. This proposition is supported by the literature, as it has been shown that IFN-α can, in fact, negatively regulate Th2 cytokine expression by inhibiting its transcription factor GATA-3.^40^

Regarding IL-10, IFN-γ and IL-6, this study found that these cytokines significantly increased after exposure of eosinophilic nasal polyp cultures to IFN-α. IL-10 is considered an important antiinflammatory cytokine and it is crucial in preventing tissue damage caused by inflammation. It is produced by a variety of cells, especially Treg and Th2, regulating both the innate and adaptive immune responses.^41^ The role of IL-10 in the physiopathogeny of eosinophilic CRSwNP is still controversial, since the authors diverge regarding the increase of IL-10 expression in polypoid tissue in relation to the sinonasal mucosa.^42^, ^43^ Recently, however, some authors have suggested that the endotype of patients with CRSwNP, characterized by the presence of IL-10, shows less severe and more easily treatable disease.^44^

In fact, it has been shown that IFN-α is able to increase IL-10 expression in activated peripheral CD4 + T cells and in whole blood culture.^45^ Thus, it is possible that IFN-α, by increasing the concentration of IL-10 in eosinophilic nasal polyp cell cultures, modulates a regulatory action on the eosinophilic inflammatory process. IFN-γ is the main Th1 cytokine secreted by CD8 + lymphocytes, NK cells, B cells and other antigen-presenting cells. The levels of this interleukin are increased in CRSsNP and decreased in CRSwNP.^46^ However, we observed a significant increase in this cytokine after stimulation of eosinophilic nasal polyp cell cultures with IFN-α. According to Zhu et al., one of the ways through which the GATA-3 transcription factor increases the development of the Th2 profile is by inhibiting the Th1 response. Also, according to the authors, this inhibitory mechanism would possibly occur by blocking Tbet expression, the main Th1 transcription factor.^47^ Therefore, since IFN-α blocks the transcription factor GATA-3,^40^ it can be inferred that it decreases negativity on Th1 development by increasing IFN-γ concentration in cell cultures. IL-6 is a multifunctional cytokine produced by several cells, including epithelial cells, B and T lymphocytes, macrophages, eosinophils, mast cells and fibroblasts. The studies have shown that this interleukin is present in polypoid tissue, although its participation in the physiopathogeny of nasal polyposis is not well defined. In the study by Danielsen et al., they evaluated 13 patients with nasal polyposis and found a significant increase in IL-6 concentration in the polypoid tissue, when compared to the inferior turbinate mucosa of the patients themselves.^48^ In another study, Peters et al. evaluated the IL-6 trans-signaling pathway in the sinus mucosa of patients with CRSsNP, in the polypoid tissue of patients with CRSwNP and in the inferior turbinate mucosa of control patients. For that purpose, the concentrations of IL-6, its soluble receptor (sIL-6R), soluble glycoprotein 130 (sgp130) and transcription factor STAT3 were measured. There was a significant increase in IL-6, sIL-6R and sgp130 in polypoid tissue when compared to mucosal extracts of patients with CRSsNP and controls. However, STAT3 concentration was significantly lower in polypoid tissue. The increase in IL-6 and sIL-6R in the polypoid tissue favors the trans-signaling pathway, unlike what occurs in the presence of a low STAT3 concentration and the increase in sgp130. According to the authors, these findings suggest that this IL-6 signaling pathway is repressed in polypoid tissue.^49^ The present study also showed a significant increase in IL-6 concentration after IFN-α stimulation in the cell cultures of eosinophilic nasal polyps. Therefore, it is reasonable to suppose that IFN-α potentiates the IL-6 signaling pathway, probably positively modulating the transcription factor STAT3. Moreover, if the IL-6 trans-signaling pathway (responsible for its proinflammatory actions) is inhibited in the polypoid tissue, it may signal via its classical pathway, thereby determining anti-inflammatory actions.^50^

## Conclusion

In summary, this study showed that IFN-α altered the cytokine pattern in cell culture supernatants of eosinophilic nasal polyp samples. Their stimulation with IFN-α resulted in a significant decrease in IL-4 concentration (Th2 profile) and a significant increase in IL-10 (regulatory profile), IFN-γ (Th1 profile) and IL-6. These observations, taken together, suggest that the action of IFN-α *in vitro* results in a rebalancing of inflammatory profiles in such cultures, favoring the expression of Th1 and regulatory cytokines over Th2 cytokines.

## Conflicts of interest

The authors declare no conflicts of interest.
